# Acute Mountain Sickness Symptoms Depend on Normobaric versus Hypobaric Hypoxia

**DOI:** 10.1155/2016/6245609

**Published:** 2016-10-25

**Authors:** Dana M. DiPasquale, Gary E. Strangman, N. Stuart Harris, Stephen R. Muza

**Affiliations:** ^1^Department of Psychiatry, Massachusetts General Hospital, Harvard Medical School, Charlestown, MA, USA; ^2^Division of Wilderness Medicine, Department of Emergency Medicine, Massachusetts General Hospital, Harvard Medical School, Boston, MA, USA; ^3^Thermal and Mountain Medicine Division, U.S. Army Research Institute of Environmental Medicine, Natick, MA, USA

## Abstract

Acute mountain sickness (AMS), characterized by headache, nausea, fatigue, and dizziness when unacclimatized individuals rapidly ascend to high altitude, is exacerbated by exercise and can be disabling. Although AMS is observed in both normobaric (NH) and hypobaric hypoxia (HH), recent evidence suggests that NH and HH produce different physiological responses. We evaluated whether AMS symptoms were different in NH and HH during the initial stages of exposure and if the assessment tool mattered. Seventy-two 8 h exposures to normobaric normoxia (NN), NH, or HH were experienced by 36 subjects. The Environmental Symptoms Questionnaire (ESQ) and Lake Louise Self-report (LLS) were administered, resulting in a total of 360 assessments, with each subject answering the questionnaire 5 times during each of their 2 exposure days. Classification tree analysis indicated that symptoms contributing most to AMS were different in NH (namely, feeling sick and shortness of breath) compared to HH (characterized most by feeling faint, appetite loss, light headedness, and dim vision). However, the differences were not detected using the LLS. These results suggest that during the initial hours of exposure (1) AMS in HH may be a qualitatively different experience than in NH and (2) NH and HH may not be interchangeable environments.

## 1. Introduction

Unacclimatized individuals rapidly traveling to high altitude are at risk for developing acute mountain sickness (AMS), an illness of nonspecific symptoms including headache, nausea, vomiting, fatigue, anorexia, and dizziness. Symptoms typically start 2–12 hours following altitude exposure [1, 2]. While AMS is not life-threatening, symptoms can be disabling, causing considerable discomfort and disrupting activity. Presence and severity of AMS are most commonly assessed with two subjective Likert-style questionnaires. The Environmental Symptoms Questionnaire (ESQ) is a 67-weighted-item inventory of expected physiological and psychological symptoms developed by the US military [3, 4]. A subset of this inventory with questions related to cerebral function (AMS-C) has been validated against the full ESQ inventory [5] and is commonly used to assess AMS [6]. The second questionnaire, developed by a consensus committee, consists of five self-reported items and is known as the Lake Louise Self-report (LLS) [7]. There is no single gold-standard assessment tool [8, 9] and, unfortunately, the two questionnaires do not always produce the same diagnosis [10].

Early work defining AMS demonstrated that the most prevalent symptoms were headache and insomnia followed by various others, depending on those investigated in the study [11, 12]. Since the development of the ESQ and LLS, though, there has been limited research examining the prevalence of symptoms within each questionnaire or between questionnaires, particularly at the beginning of hypoxia exposure. Although questionnaires assessing the presence of AMS were developed for use in hypobaric hypoxia (HH)—that is, high altitude in the mountains or in a hypobaric chamber—they have been adopted to also measure AMS under conditions of simulated high altitude using normobaric hypoxia (NH). Positive scoring of AMS based on these questionnaires led to the conclusion that AMS is present in NH as well as HH [13]. This skipped a crucial step, however: determining if the known AMS symptoms due to HH are the same as those in NH.

Traditionally, AMS has been thought to primarily be the result of hypoxia. Emerging data, however, suggests that not only hypoxia, but also the hypobaria of high altitude contributes to the development of AMS [14–18]. Recently, AMS prevalence and severity have been observed to be higher in HH than NH [2, 18]. Evidence is also mounting that the two conditions may produce different performance and physiological effects as well [14, 15, 17, 19, 20]. Despite evidence supporting this, to our knowledge, no studies have examined potential differences in symptoms in the two environments.

We hypothesized that if NH and HH have different prevalence and severities of AMS, the symptoms experienced in NH and HH may also be different. Therefore, we compared the AMS symptoms most influential in AMS diagnosis in NH versus those in HH. We also compared the symptoms reported with LLS and those reported with ESQ as the two questionnaires have different diagnostic criteria and survey both similar and different symptoms.

## 2. Materials and Methods

### 2.1. Subjects

Thirty-six healthy subjects (Table 1) volunteered and were selected after screening to participate in this study approved by the Institutional Review Boards of the Massachusetts General Hospital and US Army Research Institute of Environmental Medicine. Subjects were regular exercisers born at <2134 m, living in areas that were <1220 m, and had not traveled to areas that were >1220 m for more than 2 d in the last 2 mo. After providing written informed consent, subjects were medically cleared following a clinical exam.

### 2.2. Overall Design

As part of a larger study on physiological differences between NH and HH, each subject was randomly assigned to 2 of 6 possible groups. Groups were defined by 3 environments crossed with 2 exercise durations: that is, normobaric normoxia (NN), NH, and HH crossed with short exercise (10 min) and long exercise (60 min). This was a partial repeated-measures design; having subjects participate in all 6 conditions maximizes power but was deemed impractical from both retention and potential condition-carry-over-effects perspectives. Having each subject participate in only 1 condition (fully between-subjects design) greatly reduces power due to between-subject variability. Intermediate cases (participating in 2–5 conditions) represent compromises between power and subject retention. Statistical power was further optimized—and bias minimized—by having fully counterbalanced condition-pairs and orders, resulting in 12 exposures per condition. None of the subject characteristics were different among groups (*p* > 0.05). The different exercise durations allowed for varying AMS severities among our sample [18] without reducing power since our analysis, described below, did not separate data based on exercise duration. None of the subject characteristics were different among groups (*p* > 0.05), which were fully counterbalanced condition-pairs, orders, and sexes (Table 1).

**Figure 1:**
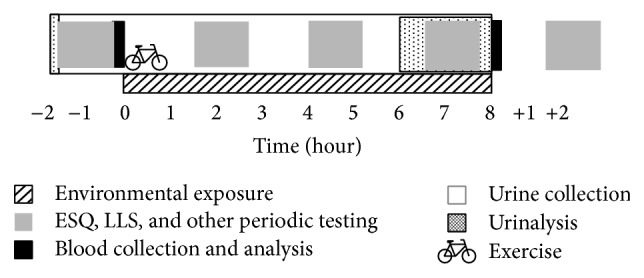
Schematic of experimental timeline. Subjects were exposed to 8 h of normobaric normoxia, normobaric hypoxia, or hypobaric hypoxia. Various physiological measurements were made before, throughout, and after testing. The ESQ and LLS were administered before exposure, 3 times over the exposure period, and after exposure.

Subjects performed sea-level testing, ascended (~15 min), exercised, spent 8 h in the environmental condition with periodic testing, and were tested again at sea level (Figure 1). Periodic testing included ~75 min battery of measurements (i.e., noninvasive cerebral and systemic physiology and cognition) including the ESQ and LLS, which were administered at the beginning of each period. Testing was performed 1.5 h before exposure, at 1.5, 4, and 6.5 h into exposure, and 1.5 h after exposure. In between testing bouts, subjects were permitted to rest, read, listen to music, or watch movies for ~75 min. Subjects were advised not to consume alcohol or exercise 24 h prior to testing. Regular coffee drinkers were permitted their usual morning beverage prior to testing. Subjects were provided food and caffeine-free drinks* ad libitum* for the remainder of the day. Two weeks separated testing days.

### 2.3. Environmental Exposures

Subjects were naive to the assigned conditions. They were not provided with any information about which room was for NN, NH, or HH, and all research personnel used supplemental oxygen regardless of the condition. NN was performed in the hypobaric chamber at barometric pressure (*P*_*B*_) = 752 mmHg, which enabled secure sealing of the chamber door, further ensuring subject naivety (partial pressure of inspired oxygen (*P*_IO_2__) = 147.3 mmHg; 300 m equivalent altitude). HH was performed in a hypobaric chamber (*P*_*B*_ = 439 mmHg; *P*_IO_2__ = 81.9 mmHg; 4400 m equivalent altitude). NH was performed at ambient pressure in a hard vinyl-sided hypoxia room (Colorado Altitude Training, Boulder, CO) with ambient oxygen partial pressure matched to the HH condition at 91.7 mmHg (*P*_*B*_ = 760 mmHg; *P*_IO_2__ = 86.1 mmHg; 4400 m equivalent altitude). Following all testing, >90% of subjects could not guess or incorrectly guessed their experimental condition.

### 2.4. Exercise

As described elsewhere [18], following ascent, subjects performed moderate exercise as a stimulus to accelerate the AMS process and vary the severity of AMS. Briefly, cycling was performed at 52.1 ± 4.4% of heart rate reserve (Excalibur Lode, Groningen, The Netherlands) and was completed within the first hour of exposure.

### 2.5. Questionnaires

Symptom presence and severity were measured using a 19-question subset of the ESQ, including all of the cerebral symptoms (AMS-C) as well as those to assess fatigue and alertness, and 4 of the 5 LLS symptoms (Table 2). The LLS question assessing sleep quality was not included since subjects did not experience a night at simulated altitude. Using the ESQ, having AMS (AMS_E_+) was defined as an AMS-C score ≥ 0.7 with current or recent “altitude” exposure [5]. Using the LLS, having AMS (AMS_L_+) was defined as the presence of a headache, at least one other symptom, a cumulative score of 3 or greater, and recent exposure to “altitude” [7]. By definition, a subject in NN—either during pre-exposure testing periods or in the NN blinded condition—cannot have AMS but can present with symptoms assessed by the questionnaire as a result of boredom, fatigue, and frustration and other symptoms related to any very long day of testing; as such, all AMS diagnoses in NN were considered AMS−, but individual symptoms' severities were still rated by subjects to account for those non-AMS-related symptoms. Out of 360 observations, there were only 2 instances when a subject in NN had a symptom score meeting the criteria for AMS+. Thus, the inclusion of unrelated symptoms allows us to accurately describe the symptoms that directly related to having AMS. AMS+/− classification was designated at each time point the questionnaires were administered, such that an individual could be AMS− early in the exposure and may not be AMS+ until later.

### 2.6. Data Analysis

Since all pre-exposure measurements were conducted in NN, they were all included in the NN analysis. Post-exposure measurements made after NH and HH were included with the NH and HH analysis, respectively.

We used classification trees to identify symptoms most influential for identifying AMS+ in NH versus HH. Decision trees are a simple but extremely useful form of multiple variable analysis used in numerous fields. In medicine, categorical and regression tree analysis (CART) employs a hierarchical selection of the most influential diagnostic criteria. For example, since the seminal work on CART [21] described a tree identifying high risk patients for myocardial infarction, hundreds of studies have used CART analysis in clinical settings investigating risk and diagnosis of heart attacks.

CART is a binary recursive partitioning method whereby data are successively split along two axes—“branches”—of the explanatory variables (i.e., presence or absence of a symptom) so that at each node the symptom is chosen that maximally distinguishes the response variable (AMS+/−) in the right and left branches [21]. Branches splitting to the right indicate the presence of the symptom, and branches to the left indicate the absence of the symptom. To implement our analysis, a tree was generated with the CART “rpart” package version 4.1-9 [22] for R version 3.1.2, which determined the symptom with the optimal first split, that being the one with the greatest gain in purity at a node (creating groups of observations that are as closely related as possible, i.e., as many AMS+ observations and fewest AMS− observations as possible or vice versa), assessed by the improvement in the Gini diversity index [21]. The generation of splits stopped when no significant decrease of the impurity was achieved or the sample size was less than *n* = 20. The analysis conducted as many splits as possible, and then 10-fold cross-validation was performed to identify the optimal model with good generalizability [23]. This was accomplished by splitting the data into 10 roughly equal parts, each containing a similar distribution for AMS+. First, 9/10 of the data was used to grow the tree, and the remaining 1/10 was used as a test sample to obtain initial estimates of the tree's error rates. This was repeated 10 times such that a different 1/10 of the data was used as the test sample. The 10 minitests were combined to form error rates for trees of each possible size. The error rates were then applied to the trees based on the entire learning sample, and the trees were pruned to the lowest possible error rate [24].

To determine if different symptoms existed depending on the environment, we created 3 classification trees: (1) all environments combined, (2) NN + NH, and (3) NN + HH. Observations in NN were included in every tree as a control for non-hypoxia-related symptoms. Three trees were created for each AMS questionnaire used (LLS or ESQ), resulting in a total of six trees. Those trees with an AMS diagnosis based on the ESQ criteria were split on the presence or absence (*not severity*) of symptoms without weighting any AMS-C symptoms, and those with a diagnosis based on LLS were split on the presence of absence of LLS symptoms.

## 3. Results

**Figure 2:**
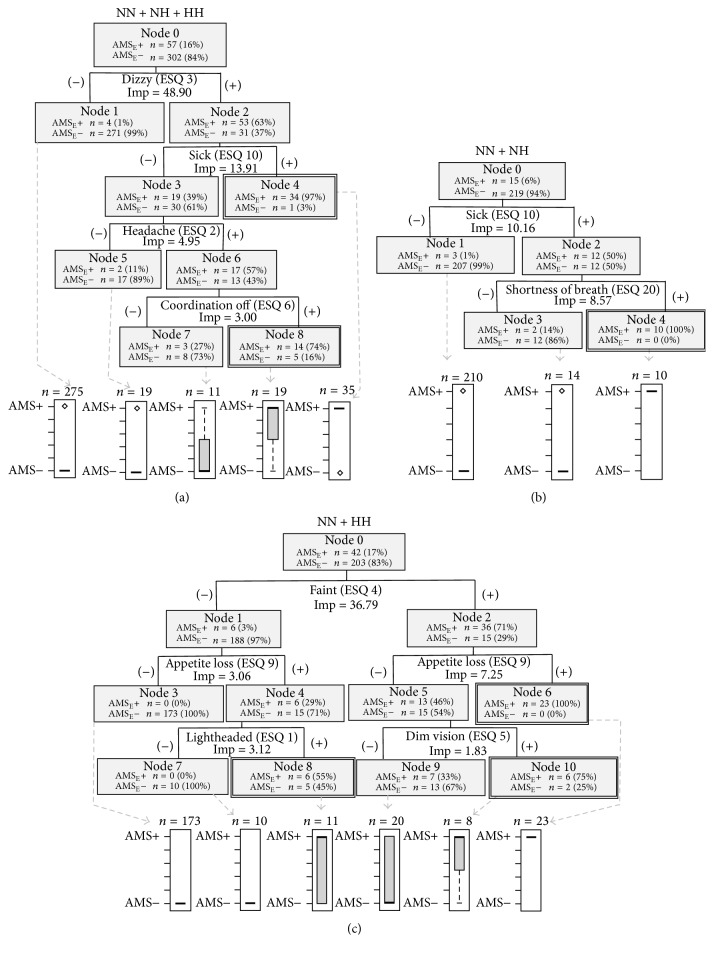
Classification trees based on ESQ symptoms and AMS_E_+ determination by AMS-C score in (a) all conditions combined, (b) NH, and (c) HH. CART analysis produces decision trees. The trees are created by splitting branch after branch. Branches were split based on the symptom listed at each node in the tree. The symptom was chosen by the CART analysis based on the importance of the symptom in determining AMS+. This importance is calculated using the Gini diversity index. If a subject had the symptom, he/she was placed in the right-hand branch. If the symptom was absent, he/she was placed in the left hand of the branch. If a majority of cases in a single node were AMS+, then the node was classified as AMS+. The double outlined terminal nodes indicate those classified as AMS_E_+ based on the box plot distributions of prediction results which are under each tree. The box plots are another graphical representation of the distribution of subjects classified closer to either AMS+ or AMS−. The findings from these trees are summarized in Table 3. Imp = importance score.

The diagrams of the tree structures created using the ESQ criteria for AMS diagnosis are presented in Figure 2. In all environmental conditions combined (Figure 2(a)), the classification tree had five terminal nodes. Two of the five nodes were classified as AMS_E_+ and three were AMS_E_− based on the distribution of AMS_E_+ and AMS_E_− observations; if the majority of observations in a node are AMS+, then the node is classified as AMS+. There were 57 observations of AMS_E_+ and 48 of them were captured in terminal nodes 4 and 8. There were 302 AMS_E_− observations, and terminal nodes 1, 5, and 7 captured 296 of them. This classification identified the following symptoms as the greatest risk factors for AMS_E_+, in order of importance: dizziness, feeling sick, headache, and coordination being off (Table 3).

In NH, AMS_E_+ was most heavily influenced by feeling sick and shortness of breath (Figure 2(b)), in order of importance (Table 3). The classification tree had 3 terminal nodes, 1 of which was classified as AMS_E_+ and the other 2 as AMS_E_−. There were 15 observations of AMS_E_+, and 10 were captured in terminal node 4. There were 219 AMS_E_− observations, and all 219 were captured in terminal nodes 1 and 3.

In HH, the classification tree had 6 terminal nodes, 3 of which were AMS_E_+ and 3 were AMS_E_− (Figure 2(c)). There were 42 observations of AMS_E_+, and 35 were captured in terminal nodes 6, 8, and 10. There were 203 AMS_E_− observations, and 196 were captured in terminal nodes 3, 7, and 9. AMS_E_+ observations in HH were most influenced by feeling faint, appetite loss, lightheadedness, and having dim vision, in order of importance (Table 3).

**Figure 3:**
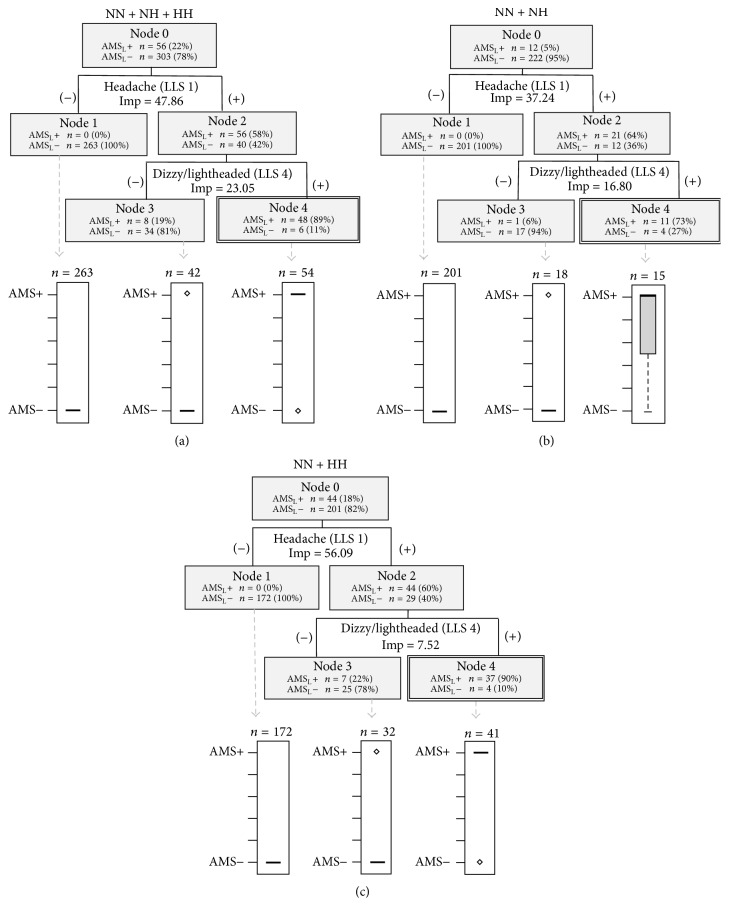
Classification trees based on LLS symptoms and AMS_L_+ determination by LLS criteria in (a) all conditions combined, (b) NH, and (c) HH. Double outlined terminal nodes indicate those classified as AMS_L_+ based on the box plot distributions of prediction results. Imp = importance score.

The diagrams of the tree structures created using the LLS criteria for AMS diagnosis are presented in Figure 3. All three classification trees had three terminal nodes. One of the three nodes was classified as AMS_L_+ and the other two were AMS_L_−. In all conditions combined, 48 of the 56 AMS_L_+ observations were captured in node 4. The other terminal nodes captured 297 of the 303 observed AMS_L_− observations. These classification trees all identified the following symptoms as the greatest risk factors for AMS_L_+, in order of importance: headache and dizzy/lightheaded (Table 3).

## 4. Discussion

We compared AMS symptoms in NH with HH, and we compared symptoms assessed by the ESQ versus LLS during the early stages of hypoxia exposure. Using a novel method for examining AMS, we progressively built up a model containing the set of symptoms that most contributed to AMS+. We found differences in NH and HH produced symptoms during an 8 h exposure, though this depended on the use of LLS or ESQ. When querying a broad set of symptoms from the ESQ, NH had fewer and different symptoms contributing to AMS+ than HH, but the LLS was not able to detect these differences. Using ESQ, AMS_E_+ in NH was characterized by feeling sick and shortness of breath, while, in HH, AMS_E_+ was primarily influenced by feeling faint, having loss of appetite, feeling lightheaded, and having dim vision. Using LLS, however, AMS_L_+ individuals experienced headache and feeling dizzy/lightheaded whether the data was separated by hypoxia condition or not.

With many potential symptoms, subjective questionnaires, different Likert scales for assessing severity, and various weights applied to symptoms, it is difficult to determine if AMS in NH is on the same continuum as AMS in HH and just less severe, or if AMS in NH is characterized by different symptoms than in HH. We performed tree analysis, a useful tool used in many fields of study, to make yes/no decisions about which symptoms are most important for classifying a condition, specifically the early stages of AMS. In many clinical models, a decision tree can have its branches split on the severity of a clinical sign, such as blood pressure >100 mmHg or body mass index >30, for example. However, using the severity of individual AMS symptoms to split branches would likely create a confusing tree that is difficult to interpret and, therefore, fail to answer the basic question—which symptoms most contribute to AMS+ in NH versus HH? Consequently, we used tree analysis by splitting branches based on the presence or absence of symptoms regardless of the severity or weight of the symptoms.

Our first tree analysis determined the symptoms most important to AMS_E_+, based on the Gini diversity index of importance, with the traditional assumption that AMS symptomatology is the same regardless of the environment. When combining all environments together, AMS_E_+ was mostly influenced by dizziness, feeling sick, having a headache, and coordination being off, in order of decreasing importance. A majority (~60%) of all AMS_E_+ observations could be correctly identified as having AMS if subjects experienced dizziness and feeling sick, and if a subject was experiencing both of these symptoms, he/she had a 97% chance of having AMS_E_+. Interestingly, not all of the identified symptoms are the most heavily weighted symptoms for determining the AMS-C score and ultimately the AMS_E_+ diagnosis. This suggests that our trees are not confounded by the differential weighting of symptoms which may have been observed if modeling trees with the severity of a symptom instead of presence/absence of a symptom.

In NH only, feeling sick and shortness of breath characterized AMS_E_+ subjects. In NH, feeling sick had the largest importance whereas, in all conditions combined, feeling sick ranked second to feeling dizzy. Subjects who did not feel sick in NH had a 99% chance of being AMS_E_−. Subjects who both felt sick and had shortness of breath had a 100% chance of having AMS_E_+. Although feeling sick is the most heavily weighted cerebral symptom, this was the only tree in which a non-AMS-C symptom—shortness of breath—was also important.

Compared to the fairly simple tree in NH using the ESQ, HH produced more branches and symptoms, including feeling faint, loss of appetite, lightheadedness, and dim vision, in order of importance. None of the symptoms in individuals with AMS_E_+ in NH were the same as those in HH, suggesting that the experience of subjects may have been qualitatively different during the initial 8 h of exposure to hypoxia. Whether or not the overall complexity of the HH tree is because of the branching decisions chosen for CART, because HH produced a more complicated illness, or because a greater AMS severity in HH was the result of numerous additive symptoms cannot be determined from this analysis. The overall sensitivity of the tree (~83%) was similar to the tree combining all conditions, suggesting the HH condition is masking the NH condition in the all-conditions-combined tree. The specificity of the HH tree was high (~97%) with many pure nodes, again showing that this method could be useful in identifying AMS_E_− subjects. Importantly, subjects who experienced both feeling faint and appetite loss had a 100% chance of having AMS_E_+. Likewise, subjects who felt faint and had dim vision had a 75% chance of having AMS_E_+. Therefore, though the overall accuracy of the tree was fair, it might be used as a quick prescreen to isolate individuals with a high likelihood of having AMS_E_+ based on just two reported symptoms. More interestingly, perhaps, HH symptoms most important in the early stages of AMS_E_+ included only cerebral symptoms, compared to NH which also included a noncerebral symptom. It is possible that a longer exposure would allow symptoms to evolve in NH and HH, such that eventually AMS symptoms in the two environments become similar. However, this remains to be determined in a controlled trial comparing NH and HH and individual symptoms.

Traditionally, AMS is thought of as a nonspecific group of symptoms with headache as a primary symptom. Our results indicated that although headache was a predictor in all-conditions-combined, environment-specific trees (NH or HH), using ESQ did not include headache as a major contributor to AMS_E_+ during the initial stages of AMS. This suggests that when combining conditions, headache may appear more important than it really is. This lends support to the camp of altitude researchers who prefer the ESQ assessment tool because it does not preclude the presence of AMS if there is no headache [25]. In contrast to ESQ, LLS requires the presence of a headache for subjects to be considered AMS_L_+ [7]. This is evident in the LLS classification trees as all had an initial split on headache, sending all headacheless subjects to a terminal AMS_L_− node. All trees, regardless of environment, indicated that headache and dizziness/lightheadedness characterized AMS_L_+. Because this characterization is different than that using a broader set of symptoms, as in the ESQ, we speculate that the LLS is not able to identify variations in AMS symptoms in the early stages of AMS. Neither dizziness nor lightheadedness was a symptom identified in NH using the ESQ, despite these symptoms being queried by both the ESQ and LLS, again suggesting LLS may not be as useful in NH as ESQ during short-term hypoxia exposure. Additionally, the use of compound symptoms in the LLS may obscure any differences as individuals who feel one symptom but not the other are unable to differentiate this on the questionnaire. Collectively, this implies that future research on early AMS symptoms in NH should be particularly cognizant of the questionnaire used for assessment.

There are three main limitations to this study. This first is that the NH and HH conditions were matched on *P*_O_2__, causing a 4.2 Torr difference in *P*_IO_2__ between hypoxic conditions. Although not possible here due to the nature of CART, in our other analyses of the physiological data collected in this study [18, 20], we used oxygen saturation (Sp_O_2__) as a covariate to account for this extremely small difference in *P*_IO_2__, since Sp_O_2__ represents the overall functional output of ventilation and pulmonary gas exchange. In all instances, we found that adding Sp_O_2__ as a covariate to our models did not markedly change the magnitude of regression coefficients or their significance, ultimately suggesting that NH and HH produced different responses. Therefore, the difference in *P*_IO_2__ between NH and HH is likely negligible. The second limitation is the cyclic nature of assessing the AMS symptoms that contribute to the AMS diagnosis when using those same symptoms to diagnose AMS. Unfortunately, though, there is currently no objective way to determine AMS+. As such, this cyclic nature is inherent. This can be observed in the findings of the LLS trees in which headache, a symptom required for AMS_L_+, was present in all trees. However, we queried numerous symptoms from the ESQ but only used the AMS-C symptoms for the AMS_E_+ classification, reducing the cyclic nature. Additionally, we divided branches of the trees based on the presence or absence of* unweighted* symptoms—not* weighted* severity scores—also reducing the cyclic nature. In fact, this was evident in our findings, in which the NH tree included non-AMS-C symptoms. Future work can further reduce the cyclic nature by using all 67 of the ESQ symptoms instead of a subset and continuing to use only the AMS-C symptoms to classify AMS_E_+. Finally, because AMS symptoms may evolve over longer durations especially after a poor night's sleep, the results of the present analysis are limited to the first third of a day of hypoxia exposure.

## 5. Conclusions

In summary, using CART to determine the symptoms that contributed most to early AMS+, we found that NH and HH did not produce common symptoms when evaluated with a range of symptoms queried by the ESQ. Conversely, using a small set of mostly compound symptoms from the LLS, these differences were not detectable. Additionally, LLS symptoms most important in classifying AMS+ were different than ESQ symptoms suggesting the questionnaires may not be interchangeable during this early time period. While our findings were based on a substantial number of data points (360), future research should (1) investigate larger data sets to see if the trees remain robust across multiple studies, (2) examine the concept that a different questionnaire specifically for NH may be more sensitive to early AMS and related symptoms, and (3) consider that the symptoms chosen to be queried may influence the definition, diagnosis, and characterization of AMS.

## Figures and Tables

**Table 1:** Subject characteristics. None of the subject characteristics were different among groups (*p* > 0.05). Data are expressed as means ± SD.

Environment	NN		NH		HH	
Exercise	10 min	60 min	10 min	60 min	10 min	60 min
Sex (*n*)	M = 6, F = 6	M = 6, F = 6	M = 6, F = 6	M = 6, F = 6	M = 5, F = 6	M = 7, F = 6
Age (y)	24.4 ± 4.2	30.6 ± 8.4	28.5 ± 10.0	25.1 ± 4.9	30.5 ± 8.3	26.9 ± 7.1
Height (cm)	172.0 ± 6.9	172.0 ± 6.4	171.5 ± 8.6	167.9 ± 10.4	170.9 ± 9.7	174.0 ± 6.6
Weight (kg)	68.7 ± 8.7	66.8 ± 8.2	68.3 ± 12.1	67.5 ± 12.0	71.3 ± 9.8	70.7 ± 10.3
HR_rest_ (bpm)	65.6 ± 7.1	62.2 ± 11.0	62.3 ± 12.6	61.3 ± 10.6	60.0 ± 9.1	62.0 ± 9.2

**Table 2:** Symptoms measured by LLS and ESQ included in the analyses.

Question	Symptom	AMS-C score weighting factor
ESQ 1	I feel lightheaded	0.489
ESQ 2	I have a headache	0.465
ESQ 3	I feel dizzy	0.446
ESQ 4	I feel faint	0.346
ESQ 5	My vision is dim	0.501
ESQ 6	My coordination is off	0.519
ESQ 7	I feel weak	0.387
ESQ 8	I feel sick to my stomach (nauseous)	0.347
ESQ 9	I lost my appetite	0.413
ESQ 10	I feel sick	0.692
ESQ 11	I feel hungover	0.584
ESQ 12	I feel tired	—
ESQ 13	I feel sleepy	—
ESQ 14	I feel thirsty	—
ESQ 15	I have a runny nose	—
ESQ 16	My vision is blurry	—
ESQ 17	My concentration is off	—
ESQ 18	My eyes feel irritated	—
ESQ 19	I am short of breath	—
LLS 1	Headache	—
LLS 2	Gastrointestinal symptoms (nausea, loss of appetite, vomiting)	—
LLS 3	Fatigue and/or weakness	—
LLS 4	Dizziness/lightheadedness	—

**Table 3:** Summary of symptoms leading to AMS+ listed in order of importance. Approximate importance scores are in parentheses.

All			NH			HH	
AMS_E_+	AMS_L_+		AMS_E_+	AMS_L_+		AMS_E_+	AMS_L_+
Dizzy (49)	Headache (48)		Sick (10)	Headache (37)		Faint (37)	Headache (56)

Sick (14)	Dizzy/lightheaded (23)		Shortness of breath (9)	Dizzy/lightheaded (17)		Appetite loss (7)	Dizzy/lightheaded (8)

Headache (5)	—		—	—		Lightheaded (3)	—

Coordination off (3)	—		—	—		Dim vision (2)	—
